# United States aid cut: alternatives for sustainable financing for health

**DOI:** 10.11604/pamj.2025.51.23.47930

**Published:** 2025-05-28

**Authors:** Gafar Bolaji Alawode, Abdul-Rahman Akintomiwa Ajibola, Michael Ajayi

**Affiliations:** 1 Development Governance International Consult, Federal Capital Territory (FCT), Abuja, Nigeria

**Keywords:** Debt-to-health swap, health financing, low- and middle-income countries, climate financing

## Abstract

Recent United States (US) foreign aid cuts, notably through Executive Order 14169 signed on January 20, 2025, have disrupted health programs in low- and middle-income countries (LMICs), threatening progress in combating HIV/AIDS, tuberculosis, and malaria. With 37 LMICs relying on US aid for over 10% of their health budgets, the funding pause has led to closures of critical health facilities, such as 200 in Afghanistan, impacting 1.84 million people. This commentary explores sustainable financing solutions, emphasizing the Debt-to-Health Swap (D2H), where debt is forgiven in exchange for health investments. Successful D2H examples include Spain´s agreements with Cameroon, Ethiopia, and the Democratic Republic of Congo, which bolstered HIV, TB, and malaria programs. Additional mechanisms like climate financing, through initiatives like the Green Climate Fund, and health taxes are proposed, though challenges like high debt servicing costs, exceeding 3% of GDP in LMICs by 2024-persist. The commentary urges LMICs and regional bodies, such as the African Union, to advocate for D2H in global forums, integrating it into debt relief discussions to enhance health system resilience. By fostering collaboration between creditors, health organizations, and governments, D2H can unlock resources for infectious diseases, maternal health, and climate-related health risks, ensuring sustainable development.

## Commentary

In recent years, substantial analyses have highlighted the significant reliance of numerous low- and middle-income countries (LMICs) on external funding, particularly from the United States, for their health programs. A key example is the U.S. President´s Emergency Plan for AIDS Relief (PEPFAR), which has played a pivotal role in the global HIV response by providing antiretroviral therapy (ART) to millions. For instance, as of September 30, 2024, PEPFAR was supporting antiretroviral therapy for 20.6 million individuals globally [1]. However, recent policy shifts have led to significant reductions in U.S. foreign aid. On January 20, 2025, President Donald Trump signed Executive Order 14169, titled “Reevaluating and Realigning United States Foreign Aid”, which mandated a pause on all U.S. foreign development assistance to assess programmatic efficiencies and alignment with U.S. foreign policy objectives. This decision has led to the termination of numerous USAID programs worldwide, severely impacting global health initiatives. The repercussions of these funding cuts are profound, especially for LMICs heavily dependent on external aid for combating priority diseases such as HIV/AIDS, tuberculosis (TB), and malaria. A study by the Center for Global Development identified 37 countries where U.S. bilateral health assistance constituted 10% or more of their government health expenditure, with 10 countries facing potential losses exceeding 50% [2]. This level of dependency underscores the vulnerability of these nations' health systems to external funding fluctuations. In Afghanistan, for example, over 200 health facilities operated by the World Health Organization have ceased operations, depriving approximately 1.84 million people of critical medical services [3]. The impact on HIV/AIDS programs is particularly alarming. Many countries rely heavily on PEPFAR for Antiretroviral Therapy (ART) provision, with some nations depending entirely on external aid for these life-saving medications. The abrupt cessation of funding threatens to reverse decades of progress in HIV prevention and treatment, potentially leading to millions of new infections and deaths.

Given the magnitude of these challenges, there is an urgent need for affected countries to prioritize domestic health financing. In recent years, innovative financing mechanisms have emerged as pivotal strategies to augment health funding in LMICs. These mechanisms aim to bridge the gap between traditional funding sources and the financial requirements necessary to achieve comprehensive health coverage. Examples include advance market commitments, development bonds, matching funds, and guarantees [4]. However, the feasibility of enhancing domestic health financing in LMICs is influenced by the varying fiscal capacities of these nations. Factors such as economic growth rates, political landscapes, taxation systems, debt levels, and budgetary priorities contribute to these disparities and influence the kind of innovative financing mechanisms, including health taxes, that are most applicable in each context. Implementing health taxes, such as levies on tobacco, alcohol, and sugary beverages, has faced resistance in several countries due to political, economic, and social factors. In Nigeria, for instance, a proposed one kobo per second telecommunication tax in 2022 to fund healthcare for vulnerable populations sparked significant public backlash, with critics arguing it would further burden citizens and questioning the equity of the approach [5]. Additionally, in many LMICs, high debt levels significantly constrain the expansion of fiscal space for health financing. Numerous nations allocate more funds to debt servicing than to their health sectors, thereby limiting resources available for healthcare improvements. Recent studies have highlighted the escalating debt servicing costs of LMICs and their impact on health expenditures. For instance, a 2023 study published in Globalization and Health indicates that external debt service in LMICs is projected to exceed 3% of GDP by 2024, surpassing public health spending during the peak of the COVID-19 pandemic in 2020 [6]. Similarly, the World Bank's International Debt Report 2024 reveals that LMICs' external debt reached a record $8.8 trillion in 2023, with debt servicing costs at an all-time high, constraining resources available for essential services like healthcare [7].

Despite the financial strain imposed by escalating debt servicing costs in low- and middle-income countries, this situation presents a strategic opportunity to leverage innovative financing mechanisms such as the Debt-to-Health Swap (D2H). D2H Swap is an innovative financing mechanism designed to address the challenges of debt burdens in LMICs, while simultaneously increasing funding for the health sector. Under this approach, a portion of a country's external debt is forgiven or reduced in exchange for the government committing to use the freed-up resources for health-related expenditures. This can include funding for health infrastructure, disease prevention programs, or the strengthening of health systems. Debt-to-Health Swap not only provides a mechanism to alleviate the debt burden of LMICs but also offers an opportunity to allocate the released funds across other critical sectors beyond health. While the primary goal of D2H is to enhance health financing, the funds can also be extended to support economic reforms that foster long-term fiscal sustainability and efficient fiscal management, thereby minimizing future debt accumulation. One of the key advantages of the D2H for lenders is that it does not always involve complete debt forgiveness. Instead, it typically involves partial debt reduction or restructuring, meaning that the lender still recovers a portion of the original loan. Moreover, this arrangement comes with specific conditions that require the borrowing country to implement fiscal and governance reforms. These reforms, including improving tax collection, enhancing public financial management, and increasing transparency in sectors such as natural resources, are designed to strengthen the country's fiscal position over time. By focusing on these reforms, D2H Swap helps ensure that the country has the necessary fiscal space to support long-term sustainable development, including in the health sector. This, in turn, improves the country´s economic resilience and ability to meet its future obligations, thereby reducing the risk of loan default. For the lender, this represents a more secure investment, as the combination of debt reduction and fiscal reform increases the likelihood of repayment for the remaining loans.

Additionally, the creditor countries leverage D2H to improve their global profile for public health interventions in the recipient country, which is often a developing country. There have been many instances where the D2H Swap has been successful. In 2017, Spain partnered with the Global Fund to Fight AIDS, Tuberculosis, and Malaria to implement debt swap agreements with Cameroon, Ethiopia, and the Democratic Republic of the Congo (DRC) [8]. These initiatives aimed to enhance human development and improve health outcomes in these African nations. Under the Debt to Health programs, significant progress was made in the health sectors of all three countries. Spain forgave Cameroon´s debt totalling €9.2 million and $16.7 million, and in exchange, Cameroon allocated €9.3 million to the Global Fund for HIV/AIDS prevention and treatment efforts [8]. Similarly, Ethiopia benefited from Spain´s cancellation of an 8.7 million debt, committing €3.2 million to the Global Fund to combat AIDS, tuberculosis, and malaria. In the DRC, Spain waived 8.3 million in debt, and the country contributed $3.4 million to the Global Fund, designated explicitly for malaria control programs [8]. These examples demonstrate how debt swaps can serve as a mechanism to bolster health systems and tackle key public health challenges in low- and middle-income countries. Another area of sustainable financing is climate financing, as climate change significantly exacerbates health risks, including the proliferation of vector-borne diseases, heat-related illnesses, and respiratory conditions due to deteriorating air quality. Despite these escalating threats, the health sector receives a disproportionately small fraction of climate financing. Notably, less than 0.5% of multilateral climate funds are allocated to health-focused projects [9].

LMICs can access climate financing as a means of sustainable development through various global initiatives. For instance, the Green Climate Fund (GCF), United Nations Development Programme (UNDP), and World Health Organization (WHO) have established the Climate and Health Co-Investment Facility. This program aims to mobilize $122 million to support developing countries in enhancing their health systems' resilience to climate impacts [10]. To effectively leverage the funding opportunities discussed, it is crucial for regional bodies and countries from LMICs to lend their voices to the call for expanded Debt-for-Health Swaps and increased access to climate financing. Regional organizations, such as the African Union (AU) and Economic Community of West African States (ECOWAS) should actively incorporate D2H into international discussions and agendas, advocating for it as a key mechanism to address both health challenges and debt burdens in LMICs. By prioritizing D2H in global forums, these bodies can help mobilize financial resources to strengthen health systems, enhance access to critical services, and mitigate the adverse effects of external debt on national health budgets. Furthermore, regional entities should work towards creating a supportive framework that encourages D2H agreements and fosters collaboration between creditor countries, international health organizations, and borrowing countries. This approach can help unlock additional resources to address pressing health needs, particularly in areas such as infectious diseases, maternal and child health, and climate-related health risks. Such advocacy will ensure that Debt-for-Health becomes a regular part of policy discussions, particularly in the context of international debt relief initiatives, and provide a more sustainable funding mechanism to enhance health system resilience in LMICs.
